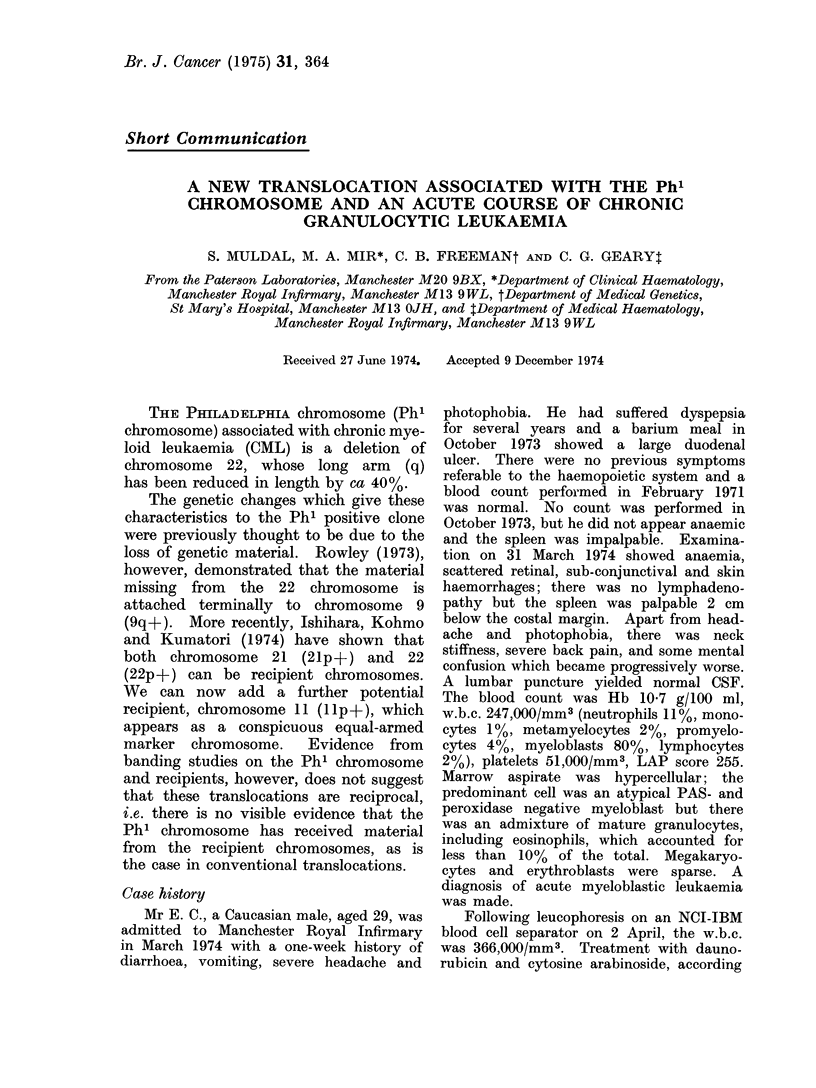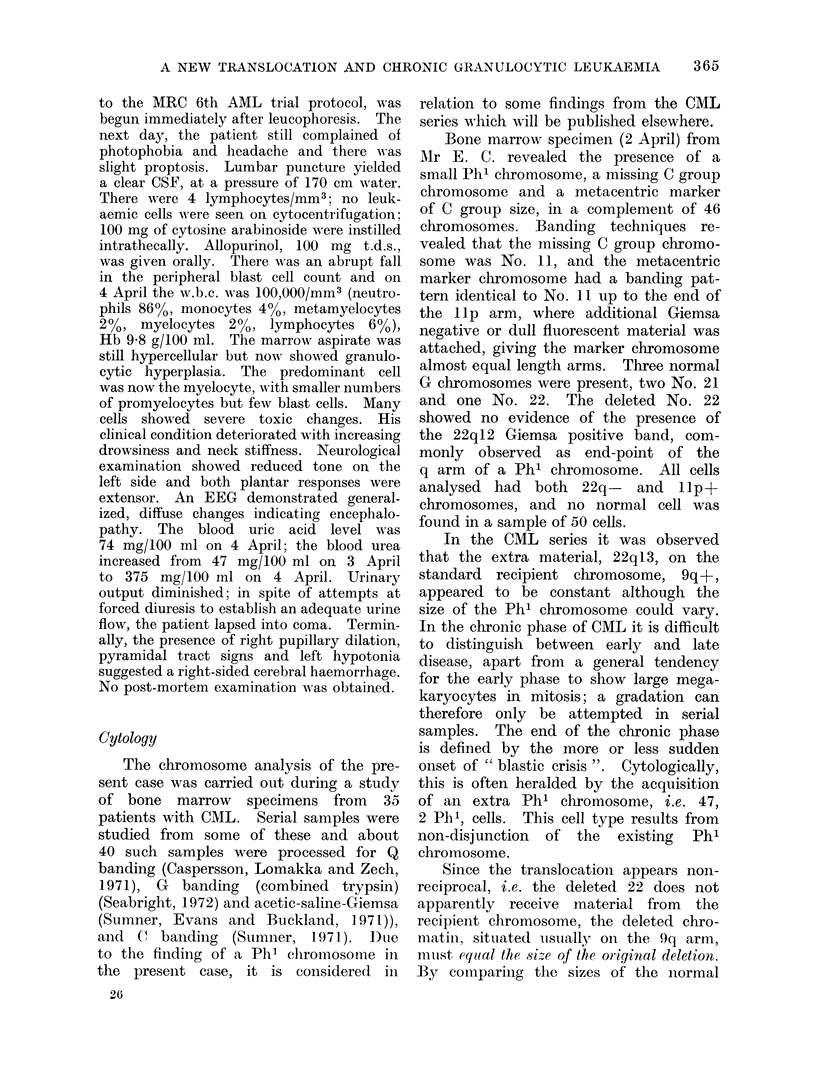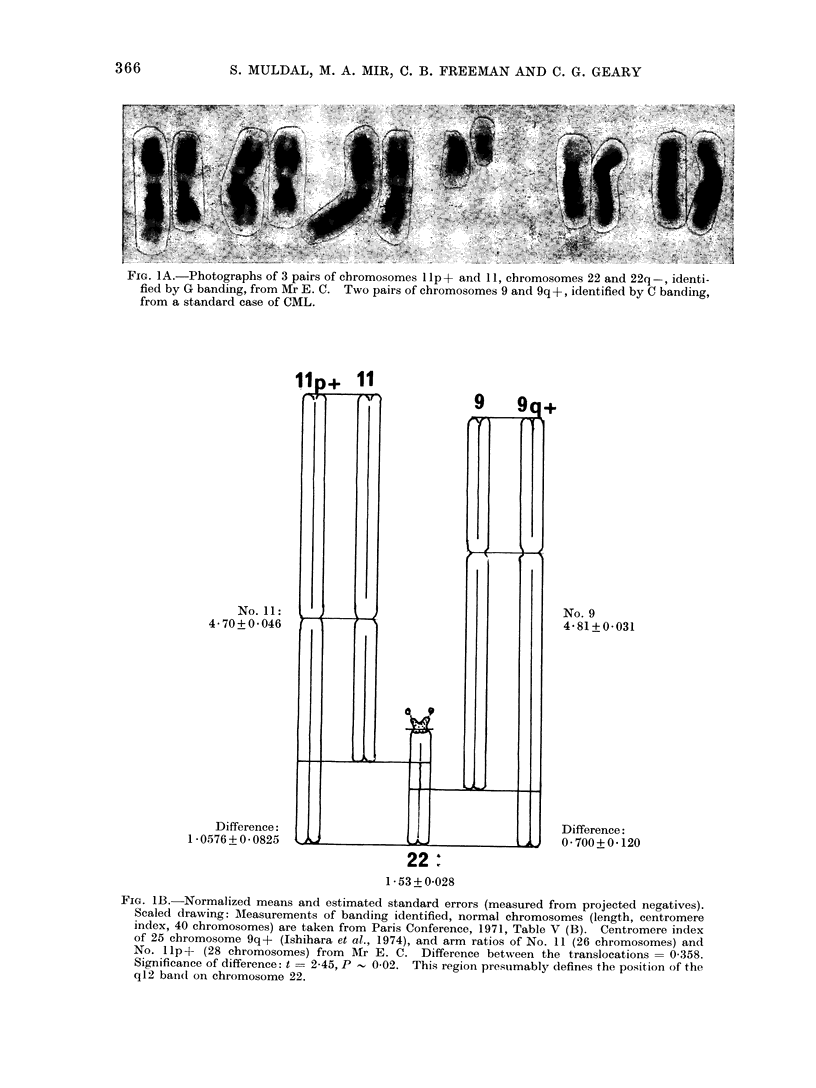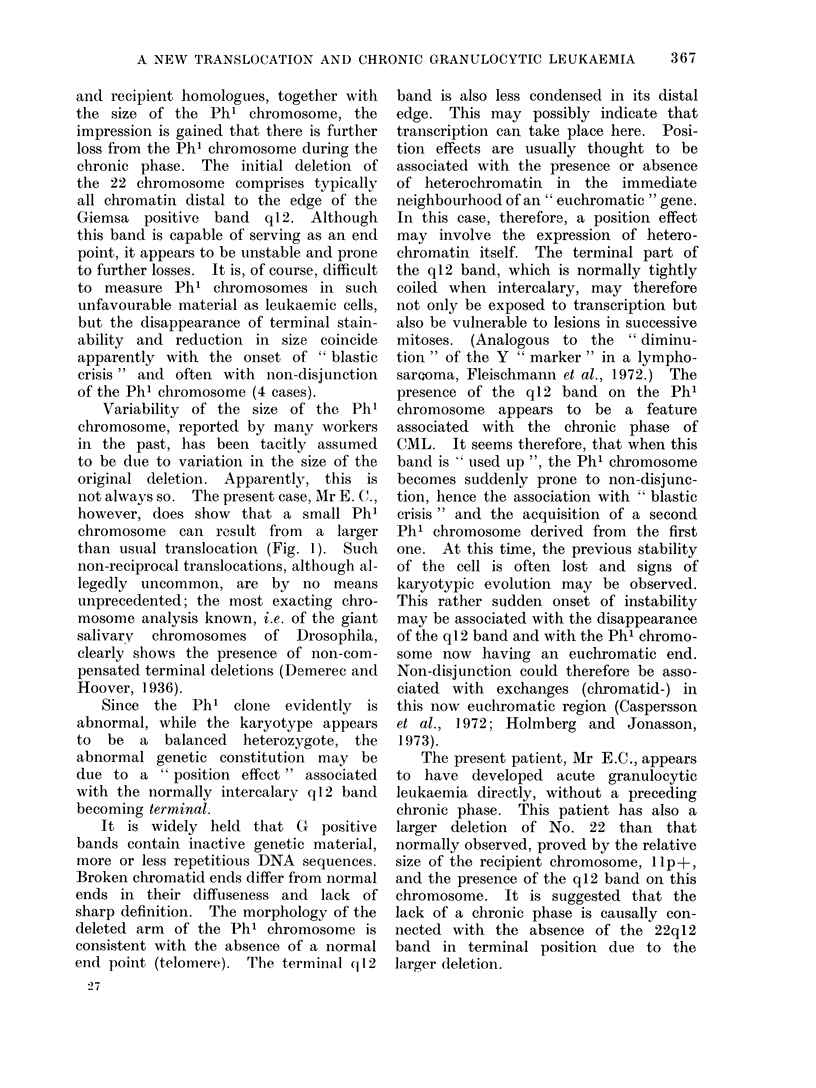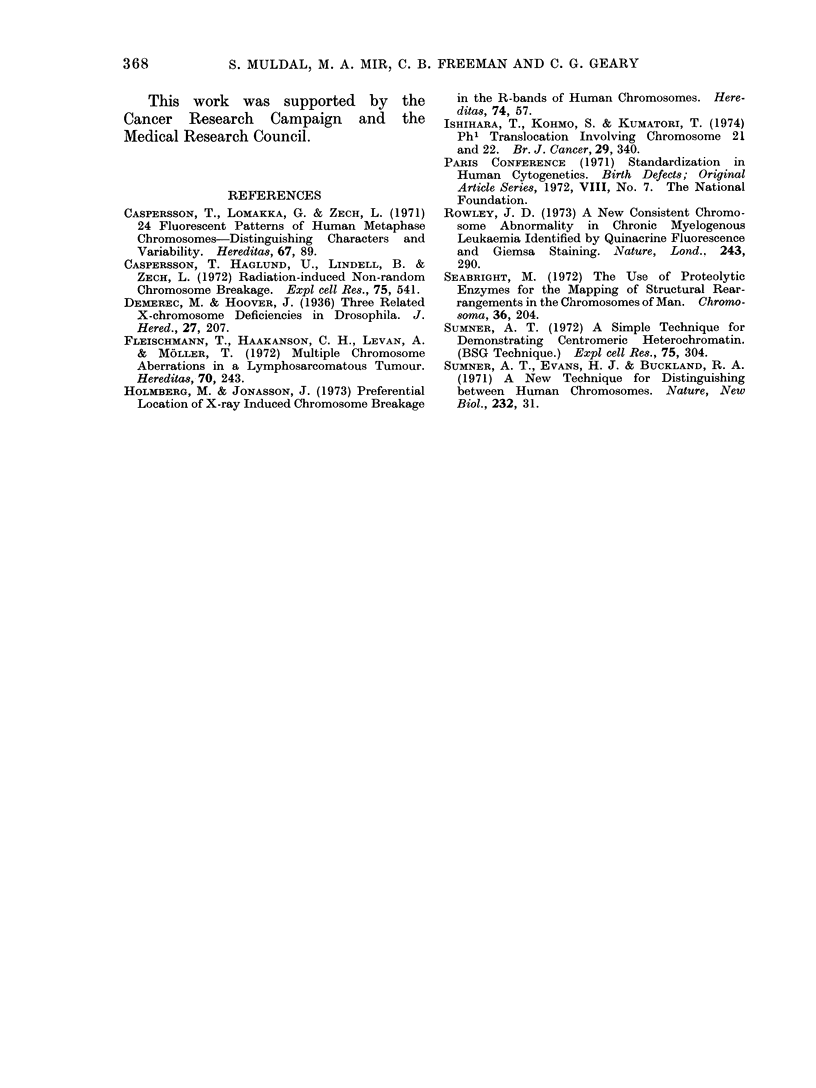# A new translocation associated with the Ph1 chromosome and an acute course of chronic granulocytic leukaemia.

**DOI:** 10.1038/bjc.1975.72

**Published:** 1975-03

**Authors:** S. Muldal, M. A. Mir, C. B. Freeman, C. G. Geary

## Abstract

**Images:**


*Br. J. Cancer* (1975) **31**, 364

***Short Communication***

### A NEW TRANSLOCATION ASSOCIATED WITH THE Ph[1] CHROMOSOME AND AN ACUTE COURSE OF CHRONIC GRANULOCYTIC LEUKAEMIA

S. MULDAL, M. A. MIR*, C. B. FREEMAN† AND C. G. GEARY‡

*From the Paterson Laboratories, Manchester M20 9BX, *Department of Clinical Haematology, Manchester Royal Infirmary, Manchester M13 9WL, †Department of Medical Genetics, St Mary's Hospital, Manchester M13 0JH, and ‡Department of Medical Haematology, Manchester Royal Infirmary, Manchester M13 9WL*

Received 27 June 1974. Accepted 9 December 1974

THE PHILADELPHIA chromosome (Ph[1] chromosome) associated with chronic myeloid leukaemia (CML) is a deletion of chromosome 22, whose long arm (q) has been reduced in length by *ca* 40%.

The genetic changes which give these characteristics to the Ph[1] positive clone were previously thought to be due to the loss of genetic material. Rowley (1973), however, demonstrated that the material missing from the 22 chromosome is attached terminally to chromosome 9 (9q+). More recently, Ishihara, Kohmo and Kumatori (1974) have shown that both chromosome 21 (21p+) and 22 (22p+) can be recipient chromosomes. We can now add a further potential recipient, chromosome 11 (11p+), which appears as a conspicuous equal-armed marker chromosome. Evidence from banding studies on the Ph[1] chromosome and recipients, however, does not suggest that these translocations are reciprocal, *i.e.* there is no visible evidence that the Ph[1] chromosome has received material from the recipient chromosomes, as is the case in conventional translocations.

### *Case history*

Mr E. C., a Caucasian male, aged 29, was admitted to Manchester Royal Infirmary in March 1974 with a one-week history of diarrhoea, vomiting, severe headache and photophobia. He had suffered dyspepsia for several years and a barium meal in October 1973 showed a large duodenal ulcer. There were no previous symptoms referable to the haemopoietic system and a blood count performed in February 1971 was normal. No count was performed in October 1973, but he did not appear anaemic and the spleen was impalpable. Examination on 31 March 1974 showed anaemia, scattered retinal, sub-conjunctival and skin haemorrhages; there was no lymphadenopathy but the spleen was palpable 2 cm below the costal margin. Apart from headache and photophobia, there was neck stiffness, severe back pain, and some mental confusion which became progressively worse. A lumbar puncture yielded normal CSF. The blood count was Hb 10·7 g/100 ml, w.b.c. 247,000/mm$^3$ (neutrophils 11%, monocytes 1%, metamyelocytes 2%, promyelocytes 4%, myeloblasts 80%, lymphocytes 2%), platelets 51,000/mm$^3$, LAP score 255. Marrow aspirate was hypercellular; the predominant cell was an atypical PAS- and peroxidase negative myeloblast but there was an admixture of mature granulocytes, including eosinophils, which accounted for less than 10% of the total. Megakaryocytes and erythroblasts were sparse. A diagnosis of acute myeloblastic leukaemia was made.

Following leucophoresis on an NCI-IBM blood cell separator on 2 April, the w.b.c. was 366,000/mm$^3$. Treatment with daunorubicin and cytosine arabinoside, according to the MRC 6th AML trial protocol, was begun immediately after leucophoresis. The next day, the patient still complained of photophobia and headache and there was slight proptosis. Lumbar puncture yielded a clear CSF, at a pressure of 170 cm water. There were 4 lymphocytes/mm$^3$; no leukaemic cells were seen on cytocentrifugation; 100 mg of cytosine arabinoside were instilled intrathecally. Allopurinol, 100 mg t.d.s., was given orally. There was an abrupt fall in the peripheral blast cell count and on 4 April the w.b.c. was 100,000/mm$^3$ (neutrophils 86%, monocytes 4%, metamyelocytes 2%, myelocytes 2%, lymphocytes 6%), Hb 9·8 g/100 ml. The marrow aspirate was still hypercellular but now showed granulocytic hyperplasia. The predominant cell was now the myelocyte, with smaller numbers of promyelocytes but few blast cells. Many cells showed severe toxic changes. His clinical condition deteriorated with increasing drowsiness and neck stiffness. Neurological examination showed reduced tone on the left side and both plantar responses were extensor. An EEG demonstrated generalized, diffuse changes indicating encephalopathy. The blood uric acid level was 74 mg/100 ml on 4 April; the blood urea increased from 47 mg/100 ml on 3 April to 375 mg/100 ml on 4 April. Urinary output diminished; in spite of attempts at forced diuresis to establish an adequate urine flow, the patient lapsed into coma. Terminally, the presence of right pupillary dilation, pyramidal tract signs and left hypotonia suggested a right-sided cerebral haemorrhage. No post-mortem examination was obtained.

### *Cytology*

The chromosome analysis of the present case was carried out during a study of bone marrow specimens from 35 patients with CML. Serial samples were studied from some of these and about 40 such samples were processed for Q banding (Caspersson, Lomakka and Zech, 1971), G banding (combined trypsin) (Seabright, 1972) and acetic-saline-Giemsa (Sumner, Evans and Buckland, 1971)), and C banding (Sumner, 1971). Due to the finding of a Ph$^1$ chromosome in the present case, it is considered in relation to some findings from the CML series which will be published elsewhere.

Bone marrow specimen (2 April) from Mr E. C. revealed the presence of a small Ph$^1$ chromosome, a missing C group chromosome and a metacentric marker of C group size, in a complement of 46 chromosomes. Banding techniques revealed that the missing C group chromosome was No. 11, and the metacentric marker chromosome had a banding pattern identical to No. 11 up to the end of the 11p arm, where additional Giemsa negative or dull fluorescent material was attached, giving the marker chromosome almost equal length arms. Three normal G chromosomes were present, two No. 21 and one No. 22. The deleted No. 22 showed no evidence of the presence of the 22q12 Giemsa positive band, commonly observed as end-point of the q arm of a Ph$^1$ chromosome. All cells analysed had both 22q− and 11p+ chromosomes, and no normal cell was found in a sample of 50 cells.

In the CML series it was observed that the extra material, 22q13, on the standard recipient chromosome, 9q+, appeared to be constant although the size of the Ph$^1$ chromosome could vary. In the chronic phase of CML it is difficult to distinguish between early and late disease, apart from a general tendency for the early phase to show large megakaryocytes in mitosis; a gradation can therefore only be attempted in serial samples. The end of the chronic phase is defined by the more or less sudden onset of " blastic crisis ". Cytologically, this is often heralded by the acquisition of an extra Ph$^1$ chromosome, *i.e.* 47, 2 Ph$^1$, cells. This cell type results from non-disjunction of the existing Ph$^1$ chromosome.

Since the translocation appears non-reciprocal, *i.e.* the deleted 22 does not apparently receive material from the recipient chromosome, the deleted chromatin, situated usually on the 9q arm, must *equal the size of the original deletion.* By comparing the sizes of the normal

FIG. 1A.—Photographs of 3 pairs of chromosomes 11p+ and 11, chromosomes 22 and 22q−, identified by G banding, from Mr E. C. Two pairs of chromosomes 9 and 9q+, identified by C banding, from a standard case of CML.

FIG. 1B.—Normalized means and estimated standard errors (measured from projected negatives). Scaled drawing: Measurements of banding identified, normal chromosomes (length, centromere index, 40 chromosomes) are taken from Paris Conference, 1971, Table V (B). Centromere index of 25 chromosome 9q+ (Ishihara *et al.*, 1974), and arm ratios of No. 11 (26 chromosomes) and No. 11p+ (28 chromosomes) from Mr E. C. Difference between the translocations = 0·358. Significance of difference: $t = 2{\cdot}45$, $P \sim 0{\cdot}02$. This region presumably defines the position of the q12 band on chromosome 22.

and recipient homologues, together with the size of the Ph[1] chromosome, the impression is gained that there is further loss from the Ph[1] chromosome during the chronic phase. The initial deletion of the 22 chromosome comprises typically all chromatin distal to the edge of the Giemsa positive band q12. Although this band is capable of serving as an end point, it appears to be unstable and prone to further losses. It is, of course, difficult to measure Ph[1] chromosomes in such unfavourable material as leukaemic cells, but the disappearance of terminal stainability and reduction in size coincide apparently with the onset of " blastic crisis " and often with non-disjunction of the Ph[1] chromosome (4 cases).

Variability of the size of the Ph[1] chromosome, reported by many workers in the past, has been tacitly assumed to be due to variation in the size of the original deletion. Apparently, this is not always so. The present case, Mr E. C., however, does show that a small Ph[1] chromosome can result from a larger than usual translocation (Fig. 1). Such non-reciprocal translocations, although allegedly uncommon, are by no means unprecedented; the most exacting chromosome analysis known, *i.e.* of the giant salivary chromosomes of Drosophila, clearly shows the presence of non-compensated terminal deletions (Demerec and Hoover, 1936).

Since the Ph[1] clone evidently is abnormal, while the karyotype appears to be a balanced heterozygote, the abnormal genetic constitution may be due to a " position effect " associated with the normally intercalary q12 band becoming *terminal.*

It is widely held that G positive bands contain inactive genetic material, more or less repetitious DNA sequences. Broken chromatid ends differ from normal ends in their diffuseness and lack of sharp definition. The morphology of the deleted arm of the Ph[1] chromosome is consistent with the absence of a normal end point (telomere). The terminal q12 band is also less condensed in its distal edge. This may possibly indicate that transcription can take place here. Position effects are usually thought to be associated with the presence or absence of heterochromatin in the immediate neighbourhood of an " euchromatic " gene. In this case, therefore, a position effect may involve the expression of heterochromatin itself. The terminal part of the q12 band, which is normally tightly coiled when intercalary, may therefore not only be exposed to transcription but also be vulnerable to lesions in successive mitoses. (Analogous to the " diminution " of the Y " marker " in a lymphosarcoma, Fleischmann *et al.*, 1972.) The presence of the q12 band on the Ph[1] chromosome appears to be a feature associated with the chronic phase of CML. It seems therefore, that when this band is " used up ", the Ph[1] chromosome becomes suddenly prone to non-disjunction, hence the association with " blastic crisis " and the acquisition of a second Ph[1] chromosome derived from the first one. At this time, the previous stability of the cell is often lost and signs of karyotypic evolution may be observed. This rather sudden onset of instability may be associated with the disappearance of the q12 band and with the Ph[1] chromosome now having an euchromatic end. Non-disjunction could therefore be associated with exchanges (chromatid-) in this now euchromatic region (Caspersson *et al.*, 1972; Holmberg and Jonasson, 1973).

The present patient, Mr E.C., appears to have developed acute granulocytic leukaemia directly, without a preceding chronic phase. This patient has also a larger deletion of No. 22 than that normally observed, proved by the relative size of the recipient chromosome, 11p+, and the presence of the q12 band on this chromosome. It is suggested that the lack of a chronic phase is causally connected with the absence of the 22q12 band in terminal position due to the larger deletion.

This work was supported by the Cancer Research Campaign and the Medical Research Council.

### REFERENCES

CASPERSSON, T., LOMAKKA, G. & ZECH, L. (1971) 24 Fluorescent Patterns of Human Metaphase Chromosomes—Distinguishing Characters and Variability. *Hereditas*, **67**, 89.

CASPERSSON, T. HAGLUND, U., LINDELL, B. & ZECH, L. (1972) Radiation-induced Non-random Chromosome Breakage. *Expl cell Res.*, **75**, 541.

DEMEREC, M. & HOOVER, J. (1936) Three Related X-chromosome Deficiencies in Drosophila. *J. Hered.*, **27**, 207.

FLEISCHMANN, T., HAAKANSON, C. H., LEVAN, A. & MÖLLER, T. (1972) Multiple Chromosome Aberrations in a Lymphosarcomatous Tumour. *Hereditas*, **70**, 243.

HOLMBERG, M. & JONASSON, J. (1973) Preferential Location of X-ray Induced Chromosome Breakage in the R-bands of Human Chromosomes. *Hereditas*, **74**, 57.

ISHIHARA, T., KOHMO, S. & KUMATORI, T. (1974) $Ph^1$ Translocation Involving Chromosome 21 and 22. *Br. J. Cancer*, **29**, 340.

PARIS CONFERENCE (1971) Standardization in Human Cytogenetics. *Birth Defects; Original Article Series*, 1972, **VIII**, No. 7. The National Foundation.

ROWLEY, J. D. (1973) A New Consistent Chromosome Abnormality in Chronic Myelogenous Leukaemia Identified by Quinacrine Fluorescence and Giemsa Staining. *Nature, Lond.*, **243**, 290.

SEABRIGHT, M. (1972) The Use of Proteolytic Enzymes for the Mapping of Structural Rearrangements in the Chromosomes of Man. *Chromosoma*, **36**, 204.

SUMNER, A. T. (1972) A Simple Technique for Demonstrating Centromeric Heterochromatin. (BSG Technique.) *Expl cell Res.*, **75**, 304.

SUMNER, A. T., EVANS, H. J. & BUCKLAND, R. A. (1971) A New Technique for Distinguishing between Human Chromosomes. *Nature, New Biol.*, **232**, 31.